# Stressful Experiences in University Predict Non-suicidal Self-Injury Through Emotional Reactivity

**DOI:** 10.3389/fpsyg.2021.610670

**Published:** 2021-04-13

**Authors:** Chloe A. Hamza, Abby L. Goldstein, Nancy L. Heath, Lexi Ewing

**Affiliations:** ^1^Department of Applied Psychology and Human Development, Ontario Institute for Studies in Education, Toronto, ON, Canada; ^2^Department of Educational and Counselling Psychology, McGill University, Montreal, QC, Canada

**Keywords:** non-suicidal self-injury, self-harm, emotional reactivity, stressful experiences, post-secondary students, emerging adults, developmental, longitudinal

## Abstract

Theoretical perspectives on non-suicidal self-injury (NSSI; direct and deliberate self-injury without lethal intent such as self-cutting or hitting) have long underscored the affective regulating properties of NSSI. Less attention has been given to the processes through which individuals choose to engage in NSSI, specifically, to regulate their distress. In the present study, we tested one theoretical model in which recent stressful experiences facilitates NSSI through emotional reactivity. Further, we tested whether the indirect link between stressful experiences and NSSI was moderated by several NSSI specific risk factors (e.g., having friends who engage in NSSI). Given the widespread prevalence of NSSI among community-based samples of adolescents and emerging adults, we surveyed 1,125 emerging adults in first-year university at a large academic institution (72% female, *M*age = 17.96, 25% with a recent history of NSSI at Time 1). Participants completed an online survey three times (assessments were 4 months apart), reporting on their recent stressful experiences in university, emotional reactivity, NSSI, as well as three NSSI specific risk factors (i.e., close friend engagement in NSSI, high self-disgust, and low fear of pain). As expected, path analysis revealed that there was a significant indirect effect of recent stressful experiences on NSSI engagement, through emotional reactivity. However, this effect was maintained across moderator analyses. These novel findings underscore the salient role of proximally occurring stressors in the prediction of NSSI among emerging adults in university, and can inform developing theoretical perspectives on NSSI.

## Introduction

Non-suicidal self-injury (NSSI), which refers to the direct and deliberate destruction or alteration of bodily tissue in the absence of lethal intent (American Psychiatric Association, 2013), is a widespread mental health concern among adolescents and emerging adults (18–25 years) (Swannell et al., 2014; Gillies et al., 2018). Although NSSI often has its onset in adolescence, a second peak period of new onset may occur during the emerging adult years (Whitlock et al., 2011; Gandhi et al., 2018). Young adults attending post-secondary school may be particularly at risk for NSSI; as many as 20–30% of university students report having engaged in NSSI (Gandhi et al., 2018; Wester et al., 2018), and as many as 10–15% of emerging adults may start engaging in NSSI for the first time during the university years (Kiekens et al., 2019). Further, there is some evidence that emerging adults in university are more likely to engage in NSSI than same-aged peers not in university (Swannell et al., 2014). Engagement in NSSI confers heightened risk for aversive outcomes among students, including academic underperformance (Kiekens et al., 2016), other mental health challenges (e.g., depressive symptoms), and suicidal behavior during the later university years (Hamza and Willoughby, 2016; Kiekens et al., 2018; Burke et al., 2019). Despite the widespread prevalence of NSSI among post-secondary students (Swannell et al., 2014; Wester et al., 2018) and mounting referrals for service on college and university campuses (Xiao et al., 2017), there is a lack of theoretically informed research on the processes through which NSSI occurs, or its associated mitigating factors, during the post-secondary years. Elucidating the processes through which NSSI is initiated and maintained, as well as identifying students most at risk, is critically important to informing theory on NSSI, as well as early NSSI prevention efforts on university campuses.

Theoretical perspectives on NSSI have long underscored the affect regulating properties of NSSI (Nock and Prinstein, 2004; Chapman et al., 2006; Klonsky and Glenn, 2009), and over a decade of research has provided strong support for the role of NSSI in the modulation of emotions (Lloyd-Richardson et al., 2007; Klonsky, 2009; Turner et al., 2012; Schoenleber et al., 2014; Victor et al., 2016; Jonsson et al., 2019). Recently this literature was consolidated in a meta-analysis; researchers found that emotion regulation was the most commonly reported motivation for NSSI engagement among individuals with a history of NSSI (Taylor et al., 2018). Findings from real-time and event-level sampling studies have yielded comparable findings, such that individuals report increases in negative affect prior to NSSI, and decreased negative affect following NSSI engagement (Hamza and Willoughby, 2015; Rodríguez-Blanco et al., 2018).

Although converging evidence demonstrates that NSSI is commonly used as an emotion regulation strategy, less attention has been paid to the processes leading up to individuals engaging in NSSI to regulate their distress. Nock's (2009, 2010) integrated model on the development and maintenance of NSSI, provides a compelling description of the distal and proximal processes through which NSSI engagement may be initiated and sustained. According to Nock, early risk factors (e.g., childhood maltreatment or early invalidating environments) predispose individuals to respond to more proximally occurring stressful or aversive affective experiences with heightened emotional reactivity. Emotional reactivity, in this context, encompasses emotional sensitivity (i.e., the tendency to respond to stressful life events with heightened negative affect), emotional intensity (i.e., the tendency to experience strong emotions), and emotional persistence (i.e., difficulty returning to a neutral emotion state following a stressor) (Nock et al., 2008). Emotional reactivity has long been underscored as a temperamental factor, shaped by early biological influences but also by environmental factors, that lead to over arousal particularly in the context of extreme stress (Strelau, 1996; Muris and Ollendick, 2005). Nock suggests that heightened emotional reactivity may be the mechanism through which stress leads to increased problem coping behavior, such as NSSI (Nock, 2009, 2010).

There is some empirical support for Nock's model, as stress exposure has been widely implicated in NSSI engagement. Specifically, exposure to early abuse and other early aversive family experiences (e.g., severe parent mental illness, domestic violence) have been shown to heighten risk for NSSI engagement in adolescence and early adulthood (Ford and Gómez, 2015; Tatnell et al., 2016; Titelius et al., 2017; Brown et al., 2018). Research on other proximally occurring or developmentally relevant stressors (e.g., stressful experiences during the transition to university) in adolescence and emerging adulthood are limited, but emerging research suggests that recent stressful experiences may also exacerbate risk for NSSI in these populations (Liu et al., 2016). For example, in two studies of community-based adolescents, researchers demonstrated that exposure to stressful experiences predicted increased risk for NSSI onset over time (Hasking et al., 2013; Voon et al., 2014). Further, recent work involving daily diary and ecological momentary assessment sampling with adult community and clinical-based samples has shown that exposure to interpersonal stressors predicts increased risk for NSSI in the short term (Kyron et al., 2018; Victor et al., 2019). Researchers have urged that studying proximal stressful life events in relation to NSSI is necessary, because exposure to recent stressors has been shown to be a key precipitating factor for other mental health concerns, such as depressive episodes and suicidality (Bagge et al., 2013; Liu et al., 2016; Paul, 2018).

Although research on exposure to stressful life events and emotional reactivity is limited, there is evidence that emotional reactivity may mediate the association between stressful life events and NSSI (Nock and Mendes, 2008; Nock, 2009). Individuals who engage in NSSI consistently self-report higher levels of emotional reactivity than individuals who do not engage in NSSI (Nock and Mendes, 2008; Smith et al., 2017; Liu et al., 2020). Further, in some lab-based studies, it has been found that individuals who engage in NSSI report greater negative affect and show heightened physiological arousal (e.g., skin conductance, startle response) following mood inductions or exposure to stressors relative to individuals who do not engage in NSSI (Nock and Mendes, 2008; Nock et al., 2008; Rinnewitz et al., 2018), although these findings have been more mixed (Hooley and Franklin, 2017). In a meta-analysis on studies of emotion dysregulation and NSSI, emotional reactivity was found to be the dimension most strongly associated with NSSI engagement (as compared to other measures of dimensions of emotion dysregulation) (You et al., 2018). Emotional reactivity also has long been strongly implicated in the development of psychological distress and other mental health concerns in previous research (Strelau, 1996; Strelau and Zawadzki, 2011). It is possible then that the experience of stressful life events leads to NSSI indirectly through heightened emotional reactivity (for a similar finding on emotional reactivity as mediator of the association between psychological disorders and NSSI–see Nock et al., 2008).

In the model on the development and maintenance of NSSI, Nock (2009, 2010) regards emotional reactivity as a general risk factor for a variety of problem behaviors (e.g., substance use, disordered eating), consistent with functionalist perspectives on problem behavior engagement (Swerdlow et al., 2020). The particularly novel aspect of Nock's model is that it outlines NSSI specific factors to explain why individuals choose NSSI when distressed as opposed to other coping behaviors. For example, an individual may be more likely to engage in NSSI when distressed if they also have friends who engage in this behavior, or are not deterred by the prospective of pain. In a more recent theoretical model of NSSI engagement, Hooley and Franklin (2017) similarly assert that there are likely NSSI specific barriers that prevent individuals from accessing the coping benefits of NSSI; in the absence of these barriers, individuals are thought to be at heightened risk. Some of these proposed absent barriers map well onto the risk factors identified by Nock (e.g., having awareness of NSSI, low self-worth, low aversion to physical pain).

In the present study, we draw on Nock's model and focus on three potential NSSI specific risk factors that may moderate associations among exposure to stressful experiences, emotional reactivity, and NSSI. First, according to Nock, individuals may choose to engage in NSSI because they have learned about or observed the behavior from others (e.g., social learning hypothesis). This hypothesis is supported by findings that adolescents and young adults who engage in NSSI are more likely to have friends who engage in NSSI than individuals who do not engage in NSSI (Hasking et al., 2013; Quigley et al., 2017). Further, research has shown that an individual's disclosure of NSSI to a friend increases the friend's risk for NSSI engagement over time (Hasking et al., 2015). It follows then that individuals may be more likely to engage in NSSI when distressed, if they have friends who also engage in this behavior. Another reason individuals may choose NSSI over other coping behaviors is because they have highly negative views toward themselves, and believe that they are deserving or worthy of self-derogation (i.e., self-punishment hypothesis). Research has consistently shown that individuals who engage in NSSI report lower levels of self-esteem and self-worth (Forrester et al., 2017), and higher levels of self-criticism than individuals without a history of NSSI (Xavier et al., 2016; Ammerman and Brown, 2018). Self-disgust has been regarded as one form of self-criticism that may be particularly relevant to NSSI, because it is thought to involve hatred toward the self, as well as self-blame (Gilbert et al., 2004; Smith et al., 2015). Finally, another risk factor that may increase risk for NSSI specifically in the context of negative emotions is low aversion to pain (i.e., the pain analgesia hypothesis) (Nock, 2009). In a recent meta-analysis on pain sensitivity and NSSI, individuals who engaged in NSSI demonstrated greater pain tolerances during lab-based tasks involving exposure to pain, and rated pain as less aversive than individuals who did not engage in NSSI (Kirtley et al., 2016; Koenig et al., 2016). Moreover, research has shown that individuals who engage in NSSI report lower fear of pain over time (Willoughby et al., 2015). These findings suggest that individuals who do not perceive pain as aversive may be more likely to engage in NSSI.

Although Nock provides a useful framework for conceptualizing the processes through which NSSI occurs, there is a paucity of theoretically-driven longitudinal examinations exploring the interaction between general risk (e.g., emotional reactivity in response to stressors) and NSSI specific risk factors (e.g., friends who engage in NSSI) in the prediction of NSSI over time. Moreover, Nock's model has yet to be applied to the study of NSSI among emerging adults in university, though exposure to stressful experiences may be particularly pronounced during this period of development (Arnett, 2015), and rates of NSSI increase during this time (Wester et al., 2018; Kiekens et al., 2019). In the present study, we utilized a three-wave longitudinal research design to examine associations among recent stressful life events in university, emotional reactivity, three NSSI specific risk factors, and NSSI behavior, in a university student sample.

Conceptual models often underscore that stressful experiences lead to mental health challenges, such as NSSI (i.e., stress sensitivity/stress exposure hypothesis), but it is also possible that NSSI may lead to increased stressful experiences for individuals (i.e., stress generation hypothesis) (Burke et al., 2015; March-Llanes et al., 2017). For example, authors have long argued that individuals who are more emotionally reactive are likely to elicit more stressful experiences from their environment (Strelau, 1996). The use of a longitudinal research design in the present study enabled us to examine the direction of effects among study variables, as well as explore emotional reactivity as a mediating factor. Elucidating the processes through which recent stressful life events may lead to heightened emotional reactivity and increase risk for NSSI (or vice versa), is essential for informing efforts to circumvent NSSI among students. Moreover, identifying who is most at risk for NSSI specifically, will inform targeted prevention and intervention efforts on college and university campuses, and extend research on theory on the development and maintenance of NSSI. We expected that consistent with Nock's model, stressful experiences in university would be associated with increased risk for NSSI through emotional reactivity (i.e., an indirect effect), and that this indirect effect would be most pronounced among those with NSSI specific risk factors.

## Materials and Methods

### Participants

In the present study, 1,125 English-speaking emerging adults at a large academic institution in Canada (72% female, 28% male, 1% other, *M*age = 17.96, SD = 0.69) completed a survey three times as part of a larger ongoing longitudinal research project. Participants completed the survey starting in September of their first-year of university, and again at 4 and 8 month follow-ups. Thirty-two percent of participants identified as East Asian, 23% identified as South Asian, 21% percent of participants identified as Caucasian, 6% identified as Arab or West Asian, and 18% identified as other, including Black, West Indian, Filipino and Latin American.

### Procedure

Students were recruited during their first month of university to participate in a study on student experiences in first-year university (the study was not advertised as a study specifically on NSSI). Students were recruited broadly across campus using printed and electronic advertisements (e.g., Facebook posting, student club websites, etc.), and in-person class announcements. Interested participants contacted the lab via phone or email, and if they were eligible (i.e., enrolled in first year, and lived in the surrounding area of the university) they were assigned a unique ID number to complete the online survey. A Qualtrics survey link was sent to participants three times (baseline, fourth month, and eighth month follow-up). As compensation, participants received a gift card for a vendor of their choice (e.g., Tim Horton's, Amazon, Cineplex Odeon, etc.) in the amount of $10 at Time 1, $15 at Time 2, and $20 at Time 3.

The study was approved by the University of Toronto's Research Ethics Board (protocol: 36254), and active informed consent was obtained from all participants at each time of assessment. Although research has consistently found that asking young adults to report on their self-injury does not have any associated iatrogenic effects (Gould et al., 2005; Whitlock et al., 2013), at each assessment participants were given a 24-hour distress line contact number, as well as a list of several local resources and supports. Participants could also access these resources anytime during the survey using a “Feeling Distressed” button. At the end of the survey, participants also completed a positive mood induction which required them to reflect on one positive event from the previous day (Seligman et al., 2005).

### Measures

#### Demographics

At Time 1, participants reported on their age in years, their gender (1 = *male*, 2 = *female, 3* = *transgender, 4* = *unsure, 5* = *prefer not to disclose, 6* = *other*), and ethnicity.

#### Recent Stressful Experiences

At each assessment point, participants reported on their recent stressful experiences using the 49-item Inventory of College Students' Recent Life Experiences (ICSRLE) (Kohn et al., 1990). Participants were asked to indicate how much each stressor (e.g., lower grades than hoped for, not enough time for sleep, conflict with friends and family) had recently been a part of their life on a scale ranging from 1 = *not at all a part of my life* to 4 = *very much a part of my life*. Ratings were averaged such that higher scores represented greater exposure to stressful experiences. The ICSRLE has demonstrated strong psychometric properties among university samples (Kohn et al., 1990; Osman et al., 1994). In the present study, Cronbach's alphas at Time 1, Time 2, and Time 3 were 0.93, 0.94, and 0.95, respectively.

#### Emotional Reactivity

At each assessment point, participants completed the Emotion Reactivity Scale (ERS) (Nock et al., 2008). This measure consists of 21 items capturing three aspects of emotion reactivity: sensitivity (eight items; “I tend to get emotional very easily”), arousal/intensity (10 items; e.g., “When I experience emotions, I feel them very strongly”), and persistence (three items; e.g., “When I am angry/upset, it takes me much longer than most people to calm down”). For each statement, participants were asked to rate their experience on a scale from 0 = *not at all like me* to 4 = *completely like me*. Ratings were averaged across all items such that higher scores represented greater emotion reactivity. The ERS has shown strong internal consistency, convergent and divergent validity, and criterion-related validity among university students with and without a history of NSSI (Nock et al., 2008; Kleiman et al., 2014). Cronbach's alphas at Time 1, Time 2, and Time 3 were 0.95, 0.95, and 0.96, respectively.

#### Non-suicidal Self-Injury

At each assessment point, participants completed an adapted version of the Inventory of Statements about Self-Injury (ISAS) (Klonsky and Glenn, 2009). The measure was limited to directly self-injurious behaviors that involved direct or deliberate destruction of bodily tissue, consistent with NSSI as defined in the DSM-5 (i.e., cutting, biting, burning, carving, severe scratching, banging or hitting self, rubbing skin against rough surfaces). Participants were asked to indicate whether they engaged in each of the behaviors listed without suicidal intent within the last four months. Consistent with previous longitudinal research on NSSI, we treated NSSI a categorical variable (the presence/absence of NSSI at each assessment) (Baetens et al., 2015; Buelens et al., 2019; Gandhi et al., 2019; Robinson et al., 2019); this normalized the NSSI variable and brought outliers back into bounds. The ISAS has been shown to have good structural and construct validity, and test re-test reliability, among university undergraduate populations (Klonsky and Glenn, 2009; Glenn and Klonsky, 2011).

#### Friend Engagement in NSSI

At Time 1, one item was used to assess whether the participant had any close friends who engaged in NSSI: “Do you have any close friends who engage non-suicidal self-injury (i.e., harming one's self on purpose without suicidal intent) such as self-cutting or burning.” This approach to assessment is comparable with other existing research on friend engagement in NSSI (Hasking et al., 2013).

#### Self-Disgust

At Time 1, self-disgust was assessed with the “disgusting self” subscale from the Self Disgust Scale (SDS) (Overton et al., 2008). Participants responded to five items (e.g., “I find myself repulsive,” “I hate myself”) on a 7-point scale ranging from 1 = *strongly agree* to 7 = *strongly disagree*. All items were averaged such that higher scores represented greater self-disgust. This measure has been shown to have good internal consistency, test-retest reliability, and concurrent validity with university students (Overton et al., 2008; Simpson et al., 2010). Cronbach's alpha was 0.81 in the present study.

#### Fear of Pain

In order to assess aversion to pain the Fear of Pain Questionnaire-9 (FPQ-9) was administered at Time 1 (McNeil and Rainwater, 1998; McNeil et al., 2017). Although we did not assess pain tolerance directly, the FPQ-9 assesses aversion to nine painful experiences (e.g., “getting a papercut on your finger,” “gulping a hot drink before it has cooled”), by asking participants to rate how fearful they are of experiencing the pain associated with each item on a scale from 1 = *not at all* to 5 = *extreme*. All items were summed to a single score, with higher values indicating higher fear of pain. The FPQ-9 is a shortened version of the FPQ-III, and has demonstrated sound internal consistency and concurrent, convergent, and divergent validity among university students (McNeil et al., 2017). Cronbach's alpha in the present study was 0.82.

### Missing Data

Missing data occurred in two primary circumstances: (1) missing data within the wave (i.e., participants did not answer all questions within the survey), and (2) missing data between waves (i.e., participants did not complete the survey for a particular wave). There was very little missing data within the wave (<1%). Overall, the study also had very strong retention across the waves: 83% of participants completed all three waves of the survey, with 10% of participants completing two waves, and 7% only completing one wave. Although participants did not differ on the primary study variables, independent samples *t*-tests revealed that participants who completed all three waves were younger and more likely to be female than participants who only completed one or two waves. Missing data was estimated using the full information maximum likelihood (FIML) method. FIML was chosen due to its ability to retain cases with missing data, therefore avoiding potentially biased parameter estimates through pairwise and listwise deletion (Schafer and Graham, 2002).

### Plan of Analysis

The associations among stress, emotional reactivity, and NSSI were examined using path analysis in Mplus 8 (Muthen and Muthen, 2017). An autoregressive cross-lagged model was tested, which included stability paths within variables across time (i.e., autoregressive paths), concurrent associations among variables within waves, and associations between variables across time (i.e., cross-lagged paths). Age and gender as assessed at Time 1 were included as covariates in all analyses, with paths from age and gender to each of the other variables at each assessment point. The weighted least square mean and variance adjusted estimator was used (WLSMV) to predict presence/absence of NSSI at each assessment (Brown, 2006). Model fit was evaluated using the comparative fit index (CFI), the root mean square of approximation (RMSEA) and the non-normed fit index (TLI) (Schreiber et al., 2006). As recommended by Hu and Bentler (1999) and Schreiber et al. (2006), CFI values >0.95, RMSEAs <0.06, and TLI values >0.95 were used, simultaneously, to indicate good model fit (Hu and Bentler, 1999; Schreiber et al., 2006).

In order to identify the best fitting model overall, a Chi-Square Difference Test of relative fit was used to test whether the pattern of associations significantly varied across time by comparing a model in which paths were unconstrained over time to a model in which paths were constrained to be equal over time (i.e., a nested model) (Muthen and Muthen, 2017). To test whether three proposed NSSI specific risk factors (i.e., having friends who engage in NSSI, having high self-disgust, having low fear of pain) moderated the association between stressful experiences and NSSI through emotional reactivity, three multi-group analyses were performed. For each proposed moderator, a model in which the paths were unconstrained across group (e.g., having friends who engaged in NSSI vs. not having friends who engaged in NSSI, having high vs. low self-disgust, having high vs. low fear of pain) was compared to a nested model in which the paths were constrained to be equal by group using the Chi-Square Difference Test of relative fit. When the Chi-Square Difference Test of relative fit statistic was non-significant, the most parsimonious model (constrained) was interpreted. To test for significant indirect effects in the final model, we report on bias-corrected bootstrapped confidence intervals, based on a sample of 5,000 bootstrap samples (Hayes, 2009).

## Results

### Preliminary Analyses

Means and standard deviations of study variables at each assessment point are provided in Table 1, and study correlations are provided in Table 2. At Times 1, 2, and 3, 25%, 23%, and 17.5% of participants reported a recent history of NSSI (i.e., NSSI within the past 4 months), respectively. The most common forms of NSSI were rubbing skin against rough surfaces, banging or hitting, and severe scratching for Wave 1, and banging and hitting, severe scratching, and biting for Times 2 and 3. On average participants reported 1–2 methods of NSSI (means of 1.53, 1.51, and 1.63, respectively). At Time 1, 24% of all participants reported having a close friend who engaged in NSSI. Independent samples *t*-tests comparing individuals who engaged in NSSI vs. those who did not engage in NSSI on the study measures at Time 1 are presented in Table 3.

**Table 1:** Means and standard deviations of study variables.

**Variable**	**M(SD)**
Age (T1)	17.96 (0.69)
Stress (T1)	1.97 (0.43)
Stress (T2)	1.98 (0.44)
Stress (T3)	1.97 (0.47)
Emotional reactivity (T1)	1.60 (0.89)
Emotional reactivity (T2)	1.59 (0.90)
Emotional reactivity (T3)	1.62 (0.94)
Self-disgust (T1)	3.08 (1.24)
Friends (T1)	0.24 (0.43)
Fear of pain (T1)	26.4 (6.73)

*T1, Time 1; T2, Time 2; T3, Time 3*.

**Table 2:** Correlations among study variables.

**Variables**	**1**.	**2**.	**3**.	**4**.	**5**.	**6**.	**7**.	**8**.	**9**.	**10**.	**11**.	**12**.	**13**.	**14**.
1. Gender	–	0.005	0.172^**^	0.142^**^	0.167^**^	0.238^**^	0.249^**^	0.273^**^	0.066^*^	0.039	0.083^*^	0.031	0.178^**^	0.013
2. Age		–	−0.059	−0.059	−0.045	−0.012	0.015	0.008	−0.01	0.013	0.004	−0.056	−0.032	−0.043
3. Stress1			–	0.701^**^	0.624^**^	0.534^**^	0.472^**^	0.479^**^	0.248^**^	0.191^**^	0.173^**^	0.451^**^	0.089^**^	0.138^**^
4. Stress2				–	0.745^**^	0.448^**^	0.531^**^	0.494^**^	0.205^**^	0.235^**^	0.174^**^	0.396^**^	0.096^**^	0.124^**^
5. Stress3					–	0.436^**^	0.449^**^	0.559^**^	0.180^**^	0.210^**^	0.177^**^	0.394^**^	0.061	0.119^**^
6. ERS1						–	0.766^**^	0.715^**^	0.263^**^	0.223^**^	0.240^**^	0.390^**^	0.183^**^	0.057
7. ERS2							–	0.790^**^	0.213^**^	0.234^**^	0.233^**^	0.361^**^	0.174^**^	0.061
8. ERS3								–	0.224^**^	0.225^**^	0.260^**^	0.376^**^	0.158^**^	0.086^**^
9. NSSI1									–	0.483^**^	0.483^**^	0.291^**^	0.016	0.178^**^
10. NSSI2										–	0.493^**^	0.227^**^	0.077^*^	0.181^**^
11. NSSI3											–	0.225^**^	0.015	0.114^**^
12. SDS1												–	0.027	0.080^**^
13. FPQ1													–	0.008
14. Peer1														–

*ERS, emotional reactivity; NSSI, non-suicidal self-injury; SDS, self-disgust; FPQ, fear of pain; peer, peer engagement in NSSI. Gender (0 = male, 1 = female); NSSI1, NSSI2, NSSI3 (0 = absence of NSSI, 1 = presence of NSSI), and Peer1 are categorical variables (0 = no, 1 = yes)*.

* *p < 0.05*.

** *p < 0.01*.

**Table 3:** Mean differences between NSSI and no NSSI groups on study measures at Time 1.

**Variable**	**Total *M(SD)***	**NSSI *M(SD)***	**No NSSI *M(SD)***	**t-statistic**	***p***
Age	17.96 (0.69)	17.95 (0.73)	17.96 (0.68)	*t*(1053) = 0.334	*p* = 0.14
Gender	0.72 (0.45)	0.77 (0.42)	0.70 (0.46)	*t*(495.96) = −2.283	*p* = 0.23
Stress	1.97 (0.43)	2.15 (0.43)	1.91 (0.40)	*t*(1110) = −8.525	*p* < 0.001
Emotional reactivity	1.60 (0.89)	2.00 (0.90)	1.46 (0.84)	*t*(1114) = −9.080	*p* < 0.001
Self-disgust	3.08 (1.24)	3.69 (1.30)	2.86 (1.14)	*t*(428.17) = −9.525	*p* < 0.001
Friends	0.24 (0.43)	0.38 (0.49)	0.20 (0.40)	*t*(409.80) = −5.478	*p* < 0.001
Fear of pain	26.4 (6.73)	26.57 (6.68)	26.34 (6.75)	*t*(476.415) = −0.487	*p =* 0.63

### Primary Results

**Figure 1:**
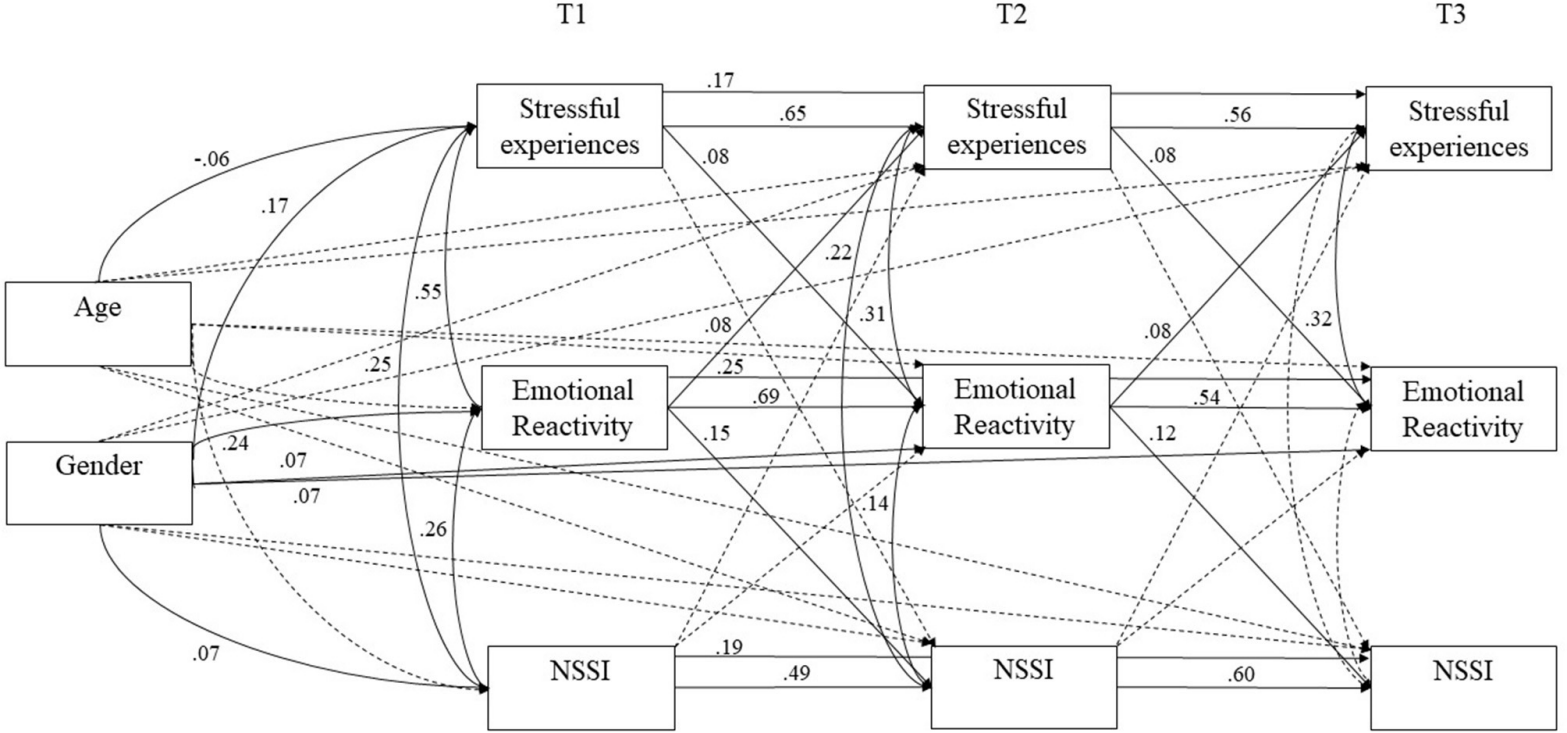
Path analysis model. T1 denotes Time 1, T2 denotes Time 2, and T3 denotes Time 3. All paths tested are included in the model; dotted lines denote non-significant paths, solid lines denote significant paths.

First, associations among stressful experiences in university, emotional reactivity, and NSSI were examined, using autoregressive cross-lagged modeling in Mplus (Muthen and Muthen, 2017). The Chi-Square Difference Test of relative fit revealed that the unconstrained model (CFI= 1.00, RMSEA = 0.012, TLI = 0.996) did not provide a significantly better fit to the constrained model, [*X*^2^(6) = 7.813], *p* = 0.25, so all further interpretations were based on the constrained model, which was more parsimonious and had good model fit (CFI = 0.999, RMSEA = 0.015, TLI = 0.995). We also ran the model with gender as a grouping variable (moderator) rather than a covariate. The Chi-Square Difference Test, [*X*^2^(6) = 4.994], *p* = 0.54, indicated that model unconstrained by gender (CFI = 0.999, RMSEA = 0.014, TLI = 0.995), did not provide significantly better fit to the model constrained for gender (CFI = 1.000, RMSEA = 0.008, TLI = 0.999), suggesting the pattern of associations did not significantly differ by gender. The final model is presented in Figure 1.

Next, three multi-group analyses were conducted to examine whether the pattern of associations varied depending on the presence of three NSSI specific risk factors (i.e., having friends who engaged in NSSI vs. not, high self-disgust vs. low self-disgust using a mean split, and high fear of pain vs. low fear of pain using a mean split). In these moderated analyses, the baseline model testing the unconstrained model by group, was compared to a model in which paths were constrained across group (e.g., having a close friend who engaged in NSSI vs. not having a close friend who engaged in NSSI). The model unconstrained for having peers who engaged in NSSI (CFI = 1.000, RMSEA = 0.000, TLI = 1.000) did not provide a significantly better fit than the unconstrained model (CFI = 1.000, RMSEA = 0.000, TLI = 1.000), [*X*^2^(6) = 6.417], *p* = 0.38. The model unconstrained for self-disgust (CFI = 1.000, RMSEA = 0.000, TLI = 1.000) did not provide a significantly better fit than the model constrained for self-disgust (CFI = 0.998, RMSEA = 0.014, TLI = 0.994), [*X*^2^(6) = 8.801], *p* = 0.19. Finally, the model unconstrained for fear of pain (CFI = 1.000, RMSEA = 0.000, TLI = 1.000) did not provide a significantly better fit than the model constrained for fear of pain (CFI = 1.000, RMSEA = 0.001, TLI = 1.000), [*X*^2^(6) = 6.912], *p* = 0.33. Findings suggest that the pattern of associations did not significantly vary based on the moderators; this was also the case when we ran the analyses using one standard deviation above or below the mean to delineate low and high risk groups. As a result we present results collapsed across groups in Table 4. Our test of indirect effects revealed that exposure to stressful experiences in university at Time 1 predicted increasing risk for NSSI at Time 3 indirectly through heightened emotional reactivity *B* = 0.010, SE = 0.003, Bootstrap CIs [0.005, 0.017], and this effect was unidirectional.

**Table 4:** Path coefficients.

**Path**	***B***	***SE***	***p***	***95% CI***
STRESS1 → STRESS2	0.653	0.019	0.000	[0.616, 0.691]
STRESS2 → STRESS3	0.563	0.023	0.000	[0.518, 0.608]
STRESS1 → STRESS3	0.171	0.022	0.000	[0.128, 0.214]
ERS1 → STRESS2	0.083	0.019	0.000	[0.046, 0.121]
ERS2 → STRESS3	0.079	0.018	0.000	[0.043, 0.114]
NSSI1 → STRESS2	0.013	0.011	0.220	[−0.008, 0.035]
NSSI2 → STRESS3	0.035	0.029	0.220	[−0.021, 0.091]
GENDER1 → STRESS2	0.011	0.025	0.654	[−0.038, 0.061]
GENDER1 → STRESS3	0.035	0.022	0.120	[−0.009, 0.079]
AGE1 → STRESS2	−0.023	0.024	0.330	[−0.070, 0.023]
AGE1 → STRESS3	−0.004	0.020	0.843	[−0.043, 0.036]
STRESS1 → ERS2	0.084	0.018	0.000	[0.050, 0.119]
STRESS2 → ERS3	0.084	0.017	0.000	[0.050, 0.118]
ERS1 → ERS2	0.694	0.019	0.000	[0.656, 0.731]
ERS2 → ERS3	0.539	0.029	0.000	[0.482, 0.596]
ERS1 → ERS3	0.247	0.028	0.000	[0.191, 0.302]
NSSI1 → ERS2	0.013	0.010	0.174	[−0.006, 0.032]
NSSI2 → ERS3	0.034	0.025	0.174	[−0.015, 0.084]
GENDER1 → ERS2	0.073	0.026	0.005	[0.022, 0.123]
GENDER1 → ERS3	0.067	0.024	0.004	[0.021, 0.114]
AGE1 → ERS2	0.028	0.017	0.090	[−0.004, 0.061]
AGE1 → ERS3	0.006	0.017	0.712	[−0.027, 0.040]
STRESS1 → NSSI2	0.013	0.034	0.709	[−0.054, 0.079]
STRESS2 → NSSI3	0.010	0.027	0.709	[−0.043, 0.063]
ERS1 → NSSI2	0.154	0.034	0.000	[0.087, 0.221]
ERS2 → NSSI3	0.119	0.027	0.000	[0.066, 0.171]
NSSI1 → NSSI2	0.487	0.030	0.000	[0.429, 0.546]
NSSI2 → NSSI3	0.597	0.051	0.000	[0.496, 0.679]
NSSI1 → NSSI3	0.188	0.043	0.000	[0.104, 0.272]
GENDER1 → NSSI2	0.003	0.044	0.951	[−0.083, 0.089]
GENDER 1 → NSSI3	0.034	0.050	0.497	[−0.065, 0.133]
AGE1 → NSSI2	0.020	0.044	0.640	[−0.065, 0.106]
AGE1 → NSSI3	0.002	0.045	0.970	[−0.087, 0.090]

*Numbers after variables indicate assessment wave; B, standardized coefficient; SE, standard error; ERS, Emotion reactivity; CI, confidence intervals*.

## Discussion

Recent research suggests that NSSI is a widespread mental health concern among emerging adults in post-secondary school (Swannell et al., 2014; Wester et al., 2018), and that the early university years may represent a period of increased risk for onset of NSSI (Gandhi et al., 2018). Despite the widespread prevalence of NSSI, little is known about the processes through which NSSI develops or is maintained during the university years. In the present study, we sought to address this gap in the literature by examining associations among stressful experiences in university, emotional reactivity, three proposed NSSI specific risk factors (i.e., friend engagement in NSSI, self-disgust and fear of pain) and NSSI. As predicted, exposure to stressful experiences was associated with increased risk for NSSI through emotional reactivity. We also anticipated that the indirect effect would be stronger for those who reported having friends who engaged in NSSI, high levels of self-disgust, and low fear of pain (relative to those without these NSSI specific risk factors). This hypothesis was not supported; the indirect effect of stressful experiences on NSSI was maintained across individuals with and without the NSSI specific risk factors.

### Stressful Experiences, Emotional Reactivity, and NSSI

The transition to university is thought to be a time of increased challenge for many students, as they encounter new stressors (e.g., living away from home for the first time, navigating new peer relationships, increased academic demands) (Arnett et al., 2014; Arnett, 2015). Although research on recently occurring and developmentally relevant stressors in relation to NSSI is limited (Liu et al., 2016), research has shown that recent or acute stressors may exacerbate risk for other mental health concerns such as depression and suicidality (Bagge et al., 2013; Paul, 2018). In line with this research, we found that greater exposure to recent stressful experiences predicted increased risk for NSSI, and this effect was unidirectional. In addition, consistent with Nock's (2009) proposed model, this relation was also indirect through emotional reactivity, which has been widely implicated in NSSI (Nock and Mendes, 2008; Nock et al., 2008; Smith et al., 2017; Liu et al., 2020). Although some researchers have argued that emotionally reactive individuals may elicit more stressful experiences from their environments (Strelau, 1996), our findings are more consistent with stress exposure models of risk, and suggest that stressful experiences may lead to heightened risk for NSSI behaviors, rather than the reverse (March-Llanes et al., 2017). Moreover, our study is the first to demonstrate that emotional reactivity may be a key mechanism to account for this association among university students.

### NSSI Specific Risk Factors

According to Nock (2009, 2010), individuals may be more likely to engage in NSSI when distressed, if they also experience NSSI specific risk factors. To our knowledge, our study is one of the first to examine the proposed associations between general risk factors for psychopathology (e.g., emotional reactivity to stressful experiences) and NSSI specific risk factors (e.g., having friends who engage in NSSI, high self-disgust, and low fear of pain) in one comprehensive model. Although we predicted that the NSSI specific risk factors would moderate the mediational pathway, this was not supported. Instead, stressful experiences predicted increased risk for NSSI indirectly through emotional reactivity across groups. There are several possible reasons that we did not find evidence of moderated mediation in the present study. One strong possibility is that the relevance of the NSSI specific risk factors varies among individuals with a history of NSSI. For example, some individuals may engage in NSSI because they have friends who engage in the behavior, but for others this risk factor may not be relevant. Indeed, in the present study 70% of individuals who engaged in NSSI at Time 1, reported they did not have any close friends who engaged in the behavior, so it is not surprising this factor did not emerge as a strong moderator. In contrast, the association between emotional reactivity in response to stressful experiences and NSSI seemed to be more relevant across the entire sample (and well-differentiated individuals with recent NSSI from those without recent NSSI). Future research using a person-centered approach to assessment, which takes into account associations among NSSI specific risk factors, could be used to explore variability in NSSI specific risk factors among those who engage in NSSI (and identify those most relevant to the majority of individuals who engage in NSSI).

Another potential explanation for our non-significant moderation is that the proposed NSSI specific risk factors may be more relevant to first time NSSI onset, rather than NSSI continuation or remittance during the university years. We examined changes in recent NSSI history, rather than first time onset NSSI. It is possible that learning about NSSI from friends may increase an individual's likelihood of trying NSSI for the first time, but that the affective reinforcing properties of NSSI maintain this behavior over time. This is consistent with research that has shown that emotion regulation (and intrapersonal motivations for engaging in NSSI) are far more prevalent than interpersonal motivations for NSSI (Taylor et al., 2018). Moreover, Hooley and Franklin (2017) have similarly argued that the motivations underlying first time NSSI engagement (e.g., trying to fit in with peers) may differ from those that sustain the behavior such as affect regulation, which is thought to be reinforced over time. In the future, researchers could specifically examine whether Nock's (2009) proposed risk factors interact with stressful experiences and emotional reactivity to predict first-time engagement.

It is also important to note that we did find group differences at baseline between students who engaged in NSSI and those who did not engage in NSSI, on two of the NSSI specific risk factors. Akin to past research, we found that individuals who engaged in NSSI were more likely to report having a friend who engaged in NSSI than individuals who did not engage in NSSI (Hasking et al., 2013; Quigley et al., 2017). Further, we also found that individuals who engaged in NSSI reported higher self-disgust than individuals without an NSSI history, similar to previous research on negative self-beliefs, self-disgust and NSSI (Smith et al., 2015; Xavier et al., 2016; Forrester et al., 2017; Ammerman and Brown, 2018). Thus, our findings on peer engagement and self-disgust are not inconsistent with previous research on risk factors for NSSI. However, our findings are novel, in that they show that even in the absence of these risk factors, the association between stressful experiences and NSSI through emotional reactivity was maintained.

Inconsistent with Nock's (2009) hypothesis that high fear and aversion to pain will deter individuals from engaging in NSSI (i.e., the pain analgesia hypothesis), individuals who engaged in NSSI did not report lower fear of pain than individuals who did not engage in NSSI. This finding seems to conflict with research that individuals who self-injure demonstrate greater pain tolerances in the lab (Kirtley et al., 2016; Koenig et al., 2016) and reduced fear of pain (Willoughby et al., 2015). However, some authors have suggested that differences in pain sensitivity observed in lab-based studies may more strongly reflect differences in willingness to endure pain, rather than measurable differences in pain sensitivity (e.g., tolerating pain because one believes they are deserving of such pain) (Hamza et al., 2014; Kirtley et al., 2016; Fox et al., 2017, 2018). In support of this contention, in one study it was found that a positive self-worth induction negated differences in willingness to endure pain between individuals who did and did not self-injure (which were present prior to the induction) (Hooley and St. Germain, 2014). Thus, it may be difficult to disentangle differences in physiological pain sensitivity from one's willingness to endure pain (i.e., the affective-cognitive component). Alternatively, it is also possible that lab and self-report measures assess different aspects of the pain experience (perceived rather than physiological), and/or that individuals may be poor at identifying their own pain aversion via self-report (Edwards and Fillingim, 2007). Future research on pain sensitivity and NSSI should examine associations between fear of pain and pain tolerance as assessed in the lab.

### Limitations and Future Directions

Despite the many strengths of the study, which include the test of a theoretically informed development model of NSSI engagement in a large sample of emerging adults, there are also several notable limitations. First, although the present study included a large sample of participants from a large academic institution in Canada, participants were predominantly female, East and South Asian, Caucasian, and from middle to upper-class family backgrounds. As a result, the present findings may not be generalizable to all university student populations, emerging adults more generally, or clinical based samples. Second, the present study utilized self-report assessments of study constructs, which are subject to recall errors (e.g., forgetting NSSI episodes). We did use comparatively shorter assessment intervals (e.g., every 4th months), than are often used in research on NSSI (e.g., annual and/or lifetime assessments). Nevertheless, future research could use more proximal assessments (e.g., shorter intervals) to capture the proposed developmental processes, or consider applying daily diary and ecological assessment approaches to capture interactions among stressful experiences, emotional reactivity, and NSSI as they occur in real time. It is possible that more recently occurring antecedents to NSSI may have stronger predictive effects, which may also account for why the moderators (assessed at Time 1) were not significant in the present analyses. Third, given the large size of the sample utilized in the present study, we chose to utilize self-report measures for key constructs; however, the study would have benefited from multiple assessments of variables, including lab-based measures. In particular, research suggests individuals may have difficulty self-reporting on their own pain sensitivity (Edwards and Fillingim, 2007); future research could also utilize experimental methods to examine differences in pain sensitivity and fear between individuals who engage in NSSI and who do not engage in NSSI.

An important extension of our work is to examine additional potential NSSI specific risk factors. In the present study we focused on three NSSI specific factors relevant to Nock's (2009) model; however, Nock and other researchers (Hooley and Franklin, 2017) have suggested that there may be other factors which could increase the likelihood an individual chooses NSSI to regulate their distress, rather than another coping behavior. For example, aversion to NSSI stimuli, such as blood or wounds, may be a strong deterrent to NSSI, even in the context of distress (Franklin et al., 2016; Hooley and Franklin, 2017). Our measure of peer influence also was specifically focused on close friend engagement. Although researchers have suggested that close friends may have the greatest influence (Quigley et al., 2017), it is also possible that participants learned of NSSI from broader peer groups or other social influences not considered in the present study (such as the media). Additionally, perceived social acceptability of NSSI may promote or hinder NSSI engagement (Hooley and Franklin, 2017); theoretically, an individual may be more likely to engage in this behavior if they think it will be accepted by close others, or if this barrier can be effectively overcome (e.g., an individual is confident they can conceal the behavior from others). Finally, Nock (2010) has also suggested that in the context of distress, individuals may choose NSSI because it's more easily accessible than some other coping behaviors (e.g., substance use). Research incorporating real time assessments of coping could better capture availability of alternatives during moments of distress.

### Conclusions and Implications

The results of the present study highlight that NSSI is a widely occurring mental health concern among emerging adults in university. Given that previous research has shown that NSSI is a robust predictor of later suicidal behavior (Kiekens et al., 2018; Muehlenkamp and Brausch, 2019), NSSI prevention should represent an important priority on college and university campuses. Our findings provide empirical support for Nock's (2009) developmental model of NSSI, and suggest that proximally occurring stressors may precipitate NSSI episodes among post-secondary students, through increased emotional reactivity (such as intense and persistent negative affect). Findings suggest that identifying effective strategies to help students manage stressors, and emotional responses to stressors during the transition to university, may also serve to reduce risk for NSSI engagement during the early university years.

## Data Availability Statement

The raw data supporting the conclusions of this article will be made available by the authors, without undue reservation.

## Ethics Statement

The studies involving human participants were reviewed and approved by University of Toronto Research Ethics Board. The patients/participants provided informed consent to participate in this study.

## Author Contributions

CH, AG, NH, and LE were involved in the conceptualization of the study, and the development of the study protocol. CH, AG, and LE were involved in participant recruitment and data collection. CH lead the data analyses with consultation from AG, LE, and NH. CH wrote the first draft of the manuscript, with all authors working on several edits of this paper. All authors contributed to the article and approved the final submitted version.

## Conflict of Interest

The authors declare that the research was conducted in the absence of any commercial or financial relationships that could be construed as a potential conflict of interest.
